# Supplementary material for: Beating Carnot efficiency with periodically driven chiral conductors
Source: Nat Commun. 2022 May 6;13:2512. doi: 10.1038/s41467-022-30039-7 (PMC9076907; doi:10.1038/s41467-022-30039-7)
Supplement: Supplementary file 1 — Supplementary Information [file 41467_2022_30039_MOESM1_ESM.pdf]

Supplementary Information: Beating Carnot efficiency with periodically driven chiral conductors

Sungguen Ryu, Rosa López, Llorenç Serra, and David Sánchez
Instituto de Física Interdisciplinar y Sistemas Complejos IFISC (CSIC-UIB), E-07122 Palma, Spain

PHOTOTRANSITION AMPLITUDE a_n

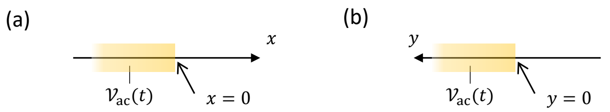

Supplementary Figure 1. Chiral channel with AC driven contact. Panel (a) is when an electron propagates out of the AC driven contact and (b) when an electron propagates into the contact.

For completeness, we summarize the derivation [26, 36] that an electron propagating in a chiral channel out of an AC driven contact [see Supplementary Figure 1 (a)] absorbs (or emits) $|n|$ photons with the transition amplitudes a_n of Eq. (2), for $n > 0$ ($n < 0$).

When an electron of initial energy $\mathcal{E}$ propagates from the AC driven contact [see Supplementary Figure 1 (a)], its wave function solution of the time-dependent Schrödinger equation is

$$\psi_{\mathcal{E}}(x,t) = e^{-i\mathcal{E}(t-x/v)}\left[\Theta(-x)e^{i\phi_{\rm ac}(t)} + \Theta(x)e^{i\phi_{\rm ac}(t-x/v)}\right]. \tag{S1}$$

Here, v is propagation velocity for the chiral channel, $\phi_{\rm ac}(t) = -e\int_{-\infty}^{t} dt'\mathcal{V}_{\rm ac}(t')/\hbar$, and $\Theta(x)$ is 0 for $x < 0$ and 1 otherwise. This solution can be verified by plugging it into the Schrödinger equation for the Hamiltonian of linear dispersion, $[i\hbar(\partial/\partial t + v\partial/\partial x) - e\mathcal{V}_{\rm ac}(t)\Theta(-x)]\psi_{\mathcal{E}}(x,t) = 0$. When an electron is in the contact, $x < 0$, the phase factor $e^{i\phi_{\rm ac}(t)}$ does not induce any change of energy as it is a spatially independent phase shift. When an electron is out of the contact, $x > 0$, the spatially dependent phase factor $e^{i\phi_{\rm ac}(t-x/v)}$ describes the electronic energy change due to the photoassisted transition. By definition of a_n in Eq. (2), $e^{i\phi_{\rm ac}(t-x/v)} = \sum_n a_n e^{-in\Omega(t-x/v)}$, thus the transition amplitude with which the electron changes its energy by $n\hbar\Omega$ is a_n.

We also remark the case when an electron propagates into the AC driven contact [see Supplementary Figure 1 (b)], which is used for the results of nonchiral conductors shown below. The wave function solution of the time-dependent Schrödinger equation with initial energy $\mathcal{E}$ is

$$\psi_{\mathcal{E}}(y,t) = e^{-i\mathcal{E}(t-y/v)}\left[\Theta(-y)+\Theta(y)e^{i\{\phi_{\rm ac}(t)-\phi_{\rm ac}(t-y/v)\}}\right]. \tag{S2}$$

This can be verified by plugging it into the Schrödinger equation $[i\hbar(\partial/\partial t + v\partial/\partial y) - e\mathcal{V}_{\rm ac}(t)\Theta(y)]\psi_{\mathcal{E}}(y,t) = 0$. According to the solution and $e^{-i\phi_{\rm ac}(t-y/v)} = \sum_n a_n^* e^{in\Omega(t-y/v)} = \sum_n a_{-n}^* e^{-in\Omega(t-y/v)}$, the transition amplitude with which the electron changes its energy by $n\hbar\Omega$ is a_{-n}^*.

UNITARITY OF FLOQUET SCATTERING MATRIX OF EQ. (3)

Here we show that the Floquet scattering matrix of Eq. (3) satisfies the unitarity conditions, $\sum_{n\alpha}|\mathcal{S}_{\alpha\beta}(\mathcal{E}_n,\mathcal{E})|^2 = 1$ and $\sum_{n\beta}|\mathcal{S}_{\alpha\beta}(\mathcal{E},\mathcal{E}_{-n})|^2 = 1$.
The first condition is satisfied as,

$$\begin{aligned}\sum_{n\alpha}|\mathcal{S}_{\alpha L}(\mathcal{E}_n,\mathcal{E})|^2 &= \sum_n |a_n|^2\Big(|t_{\rm st}(\mathcal{E}_n)|^2 + |r_{\rm st}(\mathcal{E}_n)|^2\Big)\\ &= \sum_n |a_n|^2 = 1,\end{aligned} \tag{S3}$$

$$\sum_{n\alpha}|\mathcal{S}_{\alpha R}(\mathcal{E}_n,\mathcal{E})|^2 = |t'_{\rm st}(\mathcal{E})|^2 + |r'_{\rm st}(\mathcal{E})|^2 = 1. \tag{S4}$$

The second condition is satisfied as,

$$\begin{aligned}\sum_{n\beta}|\mathcal{S}_{L\beta}(\mathcal{E},\mathcal{E}_{-n})|^2 &= \sum_n |a_n|^2|r_{\rm st}(\mathcal{E})|^2 + |t'_{\rm st}(\mathcal{E})|^2\\ &= |r_{\rm st}(\mathcal{E})|^2 + |t'_{\rm st}(\mathcal{E})|^2 = 1,\end{aligned} \tag{S5}$$

$$\begin{aligned}\sum_{n\beta}|\mathcal{S}_{R\beta}(\mathcal{E},\mathcal{E}_{-n})|^2 &= \sum_n |a_n|^2|t_{\rm st}(\mathcal{E})|^2 + |r'_{\rm st}(\mathcal{E})|^2\\ &= |t_{\rm st}(\mathcal{E})|^2 + |r'_{\rm st}(\mathcal{E})|^2 = 1.\end{aligned} \tag{S6}$$

DERIVATION OF $\langle n\rangle = 0$

Here we prove that the mean number of photons $\langle n\rangle = \sum_n n|a_n|^2$ involved in the photoassisted transitions by the AC driving is zero.

Let $a(t)$ be the Fourier transform of a_n,

$$a(t) \equiv \sum_n a_n e^{-in\Omega t}, \tag{S7}$$

$$= \exp\left[-i\frac{e}{\hbar}\int_{-\infty}^{t} dt'\,\mathcal{V}_{\rm ac}(t')\right]. \tag{S8}$$

The mean number $\langle n\rangle$ is expressed in terms of $a(t)$ as

$$\sum_n n|a_n|^2 = \frac{i}{2\pi}\int_0^{2\pi/\Omega} dt\,\frac{\partial a}{\partial t}a^*(t)\ . \tag{S9}$$

This can be verified when using Eq. (S7) and $\int_0^{2\pi/\Omega} dt\, e^{i(n-n')\Omega t} = (2\pi/\Omega)\delta_{nn'}$ for integers n and n'. Using that $|a(t')|^2 = 1$,

$$\frac{\partial a}{\partial t} a^*(t) = \frac{1}{a(t)}\frac{\partial a}{\partial t} = \frac{\partial \ln a}{\partial t} = -i\frac{e\mathcal{V}_{\rm ac}(t)}{\hbar}. \quad \text{(S10)}$$

In the last equality we used Eq. (S8). Hence, the mean number $\langle n\rangle$ is determined by the time-averaged AC voltage $\overline{\mathcal{V}_{\rm ac}}$

$$\langle n\rangle = \frac{e\overline{\mathcal{V}_{\rm ac}(t)}}{\hbar\Omega}. \quad \text{(S11)}$$

This vanishes because $\overline{\mathcal{V}_{\rm ac}} = 0$.

PHOTON NUMBER UNCERTAINTY δn IN TERMS OF AC VOLTAGE PROFILE $\mathcal{V}_{\rm ac}(t)$

Here, we derive that the photon number uncertainty δn is equal to the ratio between the root mean square of AC voltage and the photon energy quantum

$$\delta n = \frac{e\left\{\overline{\mathcal{V}^2_{\rm ac}(t)}\right\}^{1/2}}{\hbar\Omega}. \quad \text{(S12)}$$

Proof. We start by the fact that $\langle n^2\rangle$ is written in terms of $a(t)$ (see Eq. (S7)- (S8)) as

$$\sum_n n^2|a_n|^2 = -\frac{1}{2\pi\Omega}\int_0^{2\pi/\Omega} dt\,\frac{\partial^2 a}{\partial t^2}a^*(t). \quad \text{(S13)}$$

This can be verified when using Eq. (S7) and $\int_0^{2\pi/\Omega} dt\, e^{i(n-n')\Omega t} = (2\pi/\Omega)\delta_{nn'}$ for integers n and n'. Then, we use the integration by parts,

$$\sum_n n^2|a_n|^2 = -\frac{1}{2\pi\Omega}\Big[\frac{\partial a}{\partial t}a^*(t)\Big]_0^{2\pi/\Omega} + \frac{1}{2\pi\Omega}\int_0^{2\pi/\Omega} dt\,\frac{\partial a}{\partial t}\frac{\partial a^*}{\partial t}\ . \quad \text{(S14)}$$

The first term vanishes using Eq. (S10). We relate the second term to the AC voltage using $|a(t)|^2 = 1$ and

$$\frac{\partial a^*}{\partial t} = \frac{\partial}{\partial t}\frac{1}{a} = -\frac{1}{a^2}\frac{\partial a}{\partial t}. \quad \text{(S15)}$$

Using this and (S10), we obtain

$$\sum_n n^2|a_n|^2 = \frac{1}{2\pi\Omega}\int_0^{2\pi/\Omega} dt\,\Big(\frac{e\mathcal{V}_{\rm ac}(t)}{\hbar}\Big)^2. \quad \text{(S16)}$$

Then Eq. (S16) is equal to Eq. (S12) squared. □

DERIVATION OF EQ. (14)

Here we derive the deviation of entropy from the Clausius relation, Eq. (14), in the regime of small biases $k_B|\theta_L - \theta_R|$, $k_B|\mu_L - \mu_R| \ll k_B\theta_L$, and small energy uncertainty induced by the AC voltage, $\delta n\,\hbar\Omega \ll k_B\theta_L$.

First, we approximate the outgoing distribution $f^{\rm (out)}_\alpha(\mathcal{E})$ in the small driving frequency. We use the relation of the outgoing distribution to the ingoing distribution in terms of the Floquet scattering matrix,

$$f^{\rm (out)}_\alpha(\mathcal{E}) = \sum_{\beta n}|\mathcal{S}_{\alpha\beta}(\mathcal{E},\mathcal{E}_{-n})|^2 f_\beta(\mathcal{E}_{-n}). \quad \text{(S17)}$$

Due to the small energy uncertainty $\delta n\,\hbar\Omega \ll k_B\theta_L$, the ingoing distribution shifted by energy quanta (much smaller than thermal broadening) is similar to the original distribution. Therefore, we approximate

$$f_\beta(\mathcal{E}_{-n}) = f_\beta(\mathcal{E}) - n\,\hbar\Omega f'_\beta(\mathcal{E}) + \frac{(n\,\hbar\Omega)^2}{2}f''_\beta(\mathcal{E}). \quad \text{(S18)}$$

Here $f'_\beta(\mathcal{E})$ and $f''_\beta(\mathcal{E})$ are the first and second derivative of the ingoing distribution $f_\beta(\mathcal{E})$. Then, plugging Eq. (S18) into Eq. (S17), we obtain an approximation for the output distribution,

$$f^{\rm (out)}_\alpha(\mathcal{E}) = \sum_{\beta n}|\mathcal{S}_{\alpha\beta}(\mathcal{E},\mathcal{E}_{-n})|^2 f_\beta(\mathcal{E}) - \sum_{\beta n} n|\mathcal{S}_{\alpha\beta}(\mathcal{E},\mathcal{E}_{-n})|^2\,\hbar\Omega\, f'_\beta(\mathcal{E}) + \frac{1}{2}\sum_{\beta n} n^2|\mathcal{S}_{\alpha\beta}(\mathcal{E},\mathcal{E}_{-n})|^2\,(\hbar\Omega)^2\, f''_\beta(\mathcal{E})\ . \quad \text{(S19)}$$

We use Eq. (3) for the Floquet scattering matrix and sum over the photon number. Using $\langle n\rangle = 0$, we have

$$f^{\rm (out)}_\alpha(\mathcal{E}) = f^{\rm (out,st)}_\alpha(\mathcal{E}) + \frac{1}{2}(\delta n)^2\,|\mathcal{S}^{\rm (st)}_{\alpha L}(\mathcal{E})|^2\, f''_L(\mathcal{E})\,(\hbar\Omega)^2\ . \quad \text{(S20)}$$

Here, $f^{\rm (out,st)}_\alpha(\mathcal{E}) \equiv \sum_\beta |\mathcal{S}^{\rm (st)}_{\alpha\beta}(\mathcal{E})|^2 f_\beta(\mathcal{E})$ is the outgoing distribution in the static case.

From Eq. (S20), we use that the second term is much smaller than the first term, due to the condition $\delta n\,\hbar\Omega \ll k_B\theta_L$, and we approximate the Shannon entropy of the outgoing distribution up to leading order in Ω,

$$\sigma[f^{\rm (out)}_\alpha(\mathcal{E})] = \sigma[f^{\rm (out,st)}_\alpha(\mathcal{E})] + \sigma'[f^{\rm (out,st)}_\alpha(\mathcal{E})]\,\frac{(\delta n)^2}{2}\,|\mathcal{S}^{\rm (st)}_{\alpha L}(\mathcal{E})|^2\, f''_L(\mathcal{E})\,(\hbar\Omega)^2\ . \quad \text{(S21)}$$

Here $\sigma'[x] \equiv d\sigma[x]/dx$. Then, we obtain the approximation of the entropy production with the condition

$\delta n\, \hbar\Omega \ll k_B \theta_L$,

$$\overline{\dot{S}} = \overline{\dot{S}^{(\mathrm{st})}} + \sum_\alpha \frac{(\delta n)^2}{2h} \int d\mathcal{E}\, k_B\, \sigma'[f_\alpha^{(\mathrm{out,st})}(\mathcal{E})]\, |\mathcal{S}_{\alpha L}^{(\mathrm{st})}(\mathcal{E})|^2\, f_L''(\mathcal{E})(\hbar\Omega)^2, \tag{S22}$$

where $\overline{\dot{S}^{(\mathrm{st})}}$ is the time-averaged entropy production in the static case.

Now, we apply the condition of the small biases to Eq. (S22). In the leading order of the small biases, $\overline{\dot{S}^{(\mathrm{st})}} = \sum_\alpha \overline{I_h^\alpha}/\theta_\alpha$ (see below) and $\sigma'[f_\alpha^{(\mathrm{out,st})}(\mathcal{E})] \approx \sigma'[f_L^{(\mathrm{in})}(\mathcal{E})]$. Using $\sigma'[f_L^{(\mathrm{in})}(\mathcal{E})] = (\mathcal{E}-\mu_L)/(k_B\theta_L)$ and $\sum_\alpha |\mathcal{S}_{\alpha L}^{(\mathrm{st})}(\mathcal{E})|^2 = 1$, we obtain the deviation of the entropy production from the Clausius relation, in the case of small biases and small energy uncertainty

$$\delta\overline{\dot{S}} = \frac{(\delta n\, \hbar\Omega)^2}{2h\theta_L} \int d\mathcal{E}\, f_L''(\mathcal{E})(\mathcal{E}-\mu_L). \tag{S23}$$

This yields Eq. (14), because using the integration by parts, $\int d\mathcal{E}\, f_L''(\mathcal{E})(\mathcal{E}-\mu_L) = -\int d\mathcal{E}\, f_L'(\mathcal{E}) = 1$.

For completeness, we show the derivation for $\overline{\dot{S}^{(\mathrm{st})}} = \sum_\alpha \overline{I_h^\alpha}/\theta_\alpha$ in the leading order of the small biases. The outgoing distribution in the static case is expanded for small biases as,

$$\begin{aligned} f_\alpha^{(\mathrm{out,st})}(\mathcal{E}) &= \sum_\beta |S_{\alpha\beta}^{(\mathrm{st})}(\mathcal{E})|^2 f_\beta(\mathcal{E}) \\ &= f_\alpha(\mathcal{E}) + \sum_\beta |S_{\alpha\beta}^{(\mathrm{st})}(\mathcal{E})|^2 \{f_\beta(\mathcal{E}) - f_\alpha(\mathcal{E})\}\,. \end{aligned} \tag{S24}$$

Then, Shannon entropy of the outgoing distribution is expanded,

$$\sigma[f_\alpha^{(\mathrm{out,st})}(\mathcal{E})] = \sigma[f_\alpha(\mathcal{E})] + \sigma'[f_\alpha(\mathcal{E})] \sum_\beta |S_{\alpha\beta}^{(\mathrm{st})}(\mathcal{E})|^2 \{f_\beta(\mathcal{E}) - f_\alpha(\mathcal{E})\}\,. \tag{S25}$$

Using $\sigma'[f_\alpha(\mathcal{E})] = (\mathcal{E}-\mu_\alpha)/\theta_\alpha$, the entropy production is found to be,

$$\overline{\dot{S}^{(\mathrm{st})}} = \sum_{\alpha\beta} \frac{1}{h} \int d\mathcal{E}\, \frac{\mathcal{E}-\mu_\alpha}{\theta_\alpha} |S_{\alpha\beta}^{(\mathrm{st})}(\mathcal{E})|^2 \{f_\beta(\mathcal{E}) - f_\alpha(\mathcal{E})\}\,. \tag{S26}$$

This equals $\sum_\alpha \overline{I_h^\alpha}/\theta_\alpha$.

CHOICE OF DC VOLTAGE BIAS TO MAXIMIZE THE POWER

Here we discuss the choice of DC voltage bias which maximizes the generated power, for given temperatures of the reservoirs and average chemical potential μ. Then, this bias is used for obtaining the results of Figs. 2, 3 and 4. We followed the approach of the linear thermoelectricity [5], deriving response coefficients in the linear regime, namely when the thermal and voltage biases are small compared to the average temperature, $k_B|\Delta\theta|, |e\Delta V| \ll k_B\theta$, where $\Delta\theta \equiv \theta_L - \theta_R$, $\Delta V \equiv (\mu_L - \mu_R)/e$, $\theta \equiv (\theta_L + \theta_R)/2$.

To expand the generated power $-\Delta V \overline{I_e^R}$, ($\overline{P_{\mathrm{in}}} = 0$ for the setup of chiral conductors) we consider the linear regime for the current up to the first order of the biases ΔV and $\Delta\theta$. Using Eq. (7) and expanding the Fermi distributions in the small biases, we obtain $\overline{I_e^R} = \overline{I_e^R}|_{\Delta V = \Delta\theta = 0} + G\Delta V + L\Delta\theta$,

$$G = \frac{e}{h} \int d\mathcal{E}\, \frac{\mathcal{T}(\mathcal{E}) + \mathcal{T}'(\mathcal{E})}{2} \big(-f'(\mathcal{E})\big), \tag{S27}$$

$$L = \frac{1}{h} \int d\mathcal{E}\, \frac{\mathcal{T}(\mathcal{E}) + \mathcal{T}'(\mathcal{E})}{2} \big(-f'(\mathcal{E})\big) \frac{\mathcal{E}-\mu}{\theta}. \tag{S28}$$

Here, G and L are the response coefficients of the time-averaged electrical and thermoelectrical currents. $\overline{I_e^R}|_{\Delta V = \Delta\theta = 0}$ is the electric pump current in the absence of the biases. $f(\mathcal{E})$ is the Fermi-Dirac distribution of the average temperature $\theta = (\theta_L + \theta_R)/2$ and average chemical potential $\mu = (\mu_L + \mu_R)/2$. Now, the generated power is in a quadratic form of ΔV, hence generated power is maximal for the voltage bias

$$\Delta V = -\frac{L}{2G}\Delta\theta - \frac{\overline{I_e^R}|_{\Delta V = \Delta\theta = 0}}{2G}. \tag{S29}$$

PHOTOASISTED TRANSITION AMPLITUDES FOR THE LORENTZIAN VOLTAGE BIAS

For completeness, we give the photoassisted transition amplitudes a_n in the chiral conductor driven by the Lorentzian voltage pulses described in the main text, which is obtained in Ref. [36],

$$\begin{aligned} a_n &= -e^{-n\Omega w}(1 - e^{-2\Omega w})\,, \quad (n \geq 0) \\ a_{-1} &= e^{-\Omega w}\,, \\ a_n &= 0\,. \quad (n \leq -2) \end{aligned} \tag{S30}$$

DETAILED RESULTS FOR FIG. 2–3

Supplementary Figure 2 shows detailed results used to obtain Figs. 2–3.

Supplementary Figure 3 shows the outgoing distributions $f_\alpha^{(\mathrm{out})}(\mathcal{E})$ in the situation of Fig. 2, which depart from the Fermi-Dirac distribution for a fast AC driving.

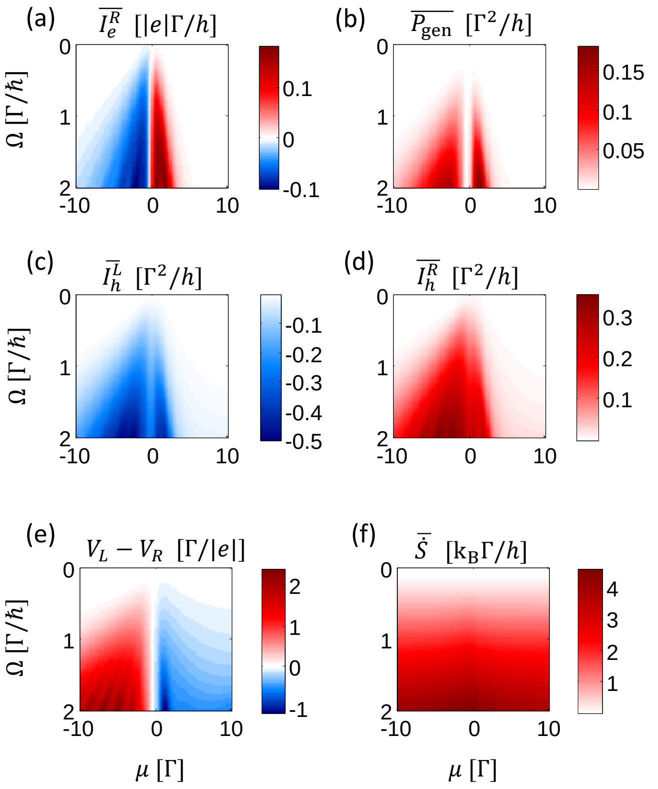

Supplementary Figure 2. Detailed results used to obtain Figs. 2 and 3, such as charge current (a), generated power (b), heat current into the left reservoir (c), heat current into the right reservoir (d), DC bias voltage choices $(\mu_L - \mu_R)/e$ maximizing generated power [see Eq. (S29)] (e), and entropy production rate (f). In all the panels, horizontal axes are average chemical potential μ in units of Γ and vertical axes are angular frequency of the ac voltage Ω in units of $\Gamma/\hbar$.

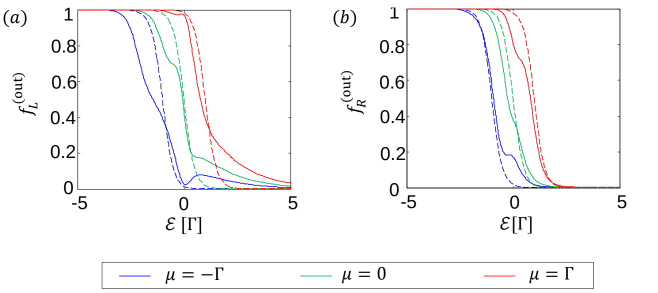

Supplementary Figure 3. The outgoing distributions $f_L^{(\rm out)}$ (a) and $f_R^{(\rm out)}$ (b) for the situation of Fig. 2. The results are shown for the AC driving frequencies of $\Omega = 1$ (solid lines) and $\Omega = 0$ (dashed lines) and the average chemical potentials $\mu = -\Gamma$ (blue), $\mu = 0$ (green), and $\mu = \Gamma$ (red).

ENGINE WITH A QUANTUM POINT CONTACT

Here we present the results of our heat engine when implemented with a quantum point contact instead of the quantum dot. The transmission probability through the quantum point contact is modeled by a step-like transmission with a single channel, $|t_{\rm st}(\mathcal{E})|^2 = [1+\exp\{-(\mathcal{E}-E_0)/\Gamma\}]^{-1}$, where E_0 is the energy at the half transmission and Γ is energy width of the step [42], see Supplementary Figure 4(b). Supplementary Figure 4(a) shows that the efficiency is also substantially enhanced, even beyond the Carnot limit. Such anomalous efficiency enhacement is acompaned by the negative entropy production $\overline{\dot{S}^{(C)}}$ given by the Clausius relation as shown in Supplementary Figure 4(c), similary to the case of the quantum dot.

We briefly discuss the differences between the case of QPC and the quantum dot. In QPC (quantum-dot) case, the efficiency is asymmetric (symmetric) for the average chemical potential μ around the half-transmission energy E_0 (resonance level). Namely, the efficiency is lower when $\mu > E_0$ when compared to the values for $\mu < E_0$. This is because the electric conductance is larger for $\mu > E_0$, and hence i) the heat extracted from the hot reservoir is larger (due to increased thermal conductance) and ii) the choice of voltage bias for maximum power is lower (we recall that such voltage bias is determined by half of the thermoelectric coefficient divided by the electric conductance in the absence of the AC driving [5]).

DERIVATION OF EQ. (15)

Here we derive Eq. (15), the electric pumping current in the regime of $\hbar\Omega \ll k_B\theta \ll \Gamma$ and $\theta_L = \theta_R = \theta$. From Eq. (7), the current becomes

$$\overline{I_e^R}_{\Delta V=\Delta\theta=0} = e\int \frac{d\mathcal{E}}{h}\{\mathcal{T}(\mathcal{E}) - \mathcal{T}'(\mathcal{E})\}f(\mathcal{E}) \tag{S31}$$

$$= e\int \frac{d\mathcal{E}}{h}\Big\{\sum_n |a_n|^2 |t_{\rm st}(\mathcal{E}_n)|^2 - |t'_{\rm st}(\mathcal{E})|^2\Big\}f(\mathcal{E}) \tag{S32}$$

Using $|t'_{\rm st}(\mathcal{E})|^2 = |t_{\rm st}(\mathcal{E})|^2$, making an expansion in $\delta n\,\hbar\Omega/(k_B\theta)$ up to second order, $\sum_n |a_n|^2 = 1$, $\sum_n n|a_n|^2 = 0$, and integrating by parts, we obtain

$$\overline{I_e^R}_{\Delta V=\Delta\theta=0} = \frac{e}{2}(\delta n\,\hbar\Omega)^2 \int \frac{d\mathcal{E}}{h}\frac{d|t_{\rm st}|^2}{d\mathcal{E}}\big(-f'(\mathcal{E})\big). \tag{S33}$$

For temperatures much lower than the energy scale where transmission varies ($k_B\theta \ll \Gamma$), the Fermi function derivative can be replaced with a Delta funcion centered at $\mathcal{E} = \mu$ and then Eq. (15) follows.

NONCHIRAL CONDUCTORS

Here, we show that for nonchiral conductors, a large power injection $\overline{P}_{\rm in}$ from the AC driving into the system diminishes the heat engine efficiency, in contrast to the

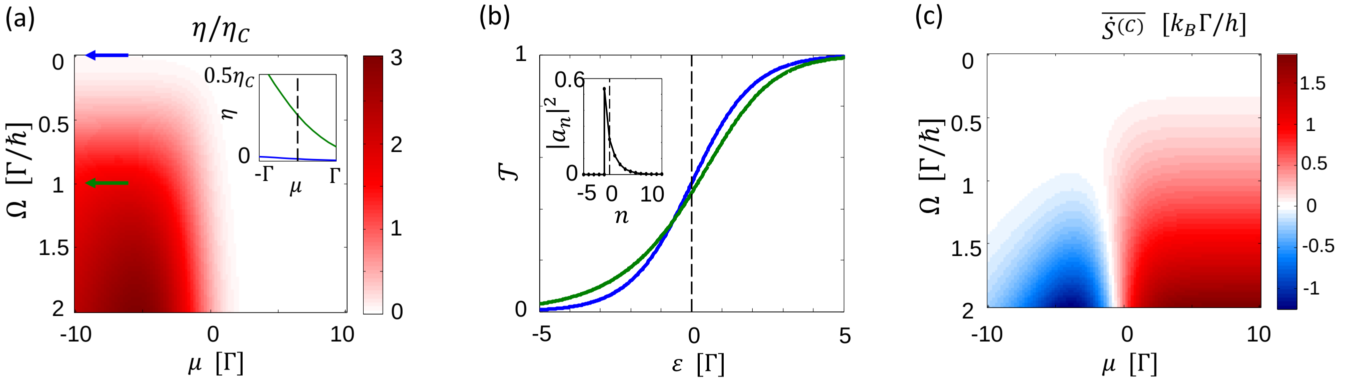

Supplementary Figure 4. Heat engine with a quantum point contact. The same situation as in Fig. 2 substituting the quantum dot to a quantum point contact is considered. (a) Efficiency when tuning the driving frequency Ω and the average chemical potential $\mu \equiv (\mu_L + \mu_R)/2$ (measured from E_0). Inset: Plot near $\mu = 0$ for $\Omega = 0$ (blue) and $\Omega = \Gamma/\hbar$ (green) as indicated with arrows in the main panel. (b) Total photoassisted transmission probability $\mathcal{T}$ for an electron of energy $\mathcal{E}$ (measured from E_0) incoming from the left reservoir for $\Omega = 0$ (blue) and $\Omega = \Gamma/\hbar$ (green). Inset: the photoassisted transition probabilities $|a_n|^2$. (c) The entropy production rate assuming Clausius relation, $\overline{\dot{S}^{(C)}}$. Here, $(\theta_L + \theta_R)/2 = 0.25\Gamma/k_B$, $\theta_L - \theta_R = 0.1\theta$, $w = 0.05 \times 2\pi/\Omega$, and the DC chemical potential bias is chosen for the maximal power generation.

case of chiral conductors. By analyzing the mean number of photons involved in the photoassisted scattering, we show that the AC power injection is positive and determined by Joule's law in the slow driving and zero temperature regime. We also show numerical results proving the efficiency decrease.

Supplementary Figure 5 shows the setup of nonchiral conductors driven by AC voltage bias. In this case, the Floquet scattering matrix (including the photoassisted processes due to the AC voltage) becomes different from that of the chiral case, due to an additional process that an electron absorbs ($n > 0$) or emits ($n < 0$) $|n|$ photons when it is back scattered into the left contact with transition amplitude a^*_{-n}; see Supplementary Figure 1 and related text. Therefore, the Floquet scattering matrix becomes

$$\begin{aligned} S_{RL}(\mathcal{E}_n, \mathcal{E}) &= a_n t_{\rm st}(\mathcal{E}_n), \\ S_{LL}(\mathcal{E}_n, \mathcal{E}) &= \sum_m a_m a^*_{-n+m} r_{\rm st}(\mathcal{E}_m), \\ S_{LR}(\mathcal{E}_n, \mathcal{E}) &= a^*_{-n} t'_{\rm st}(\mathcal{E}), \\ S_{RR}(\mathcal{E}_n, \mathcal{E}) &= \delta_{n0} r'_{\rm st}(\mathcal{E}). \end{aligned} \tag{S34}$$

Note that the reflection amplitude $S_{LL}(\mathcal{E}_n, \mathcal{E})$ from left input channel is determined by three steps rather than the two steps of the chiral case (see Eq. (3)), due to the additional photon absorption/emission process which occurs when the electron finally enters the left reservoir. As before, the Floquet scattering matrix satisfies the unitarity condition $\sum_{n\alpha} |S_{\alpha\beta}(\mathcal{E}_n, \mathcal{E})|^2 = 1$ and $\sum_{n\beta} |S_{\alpha\beta}(\mathcal{E}, \mathcal{E}_{-n})|^2 = 1$.

The power injection from AC driving $\overline{P}_{\rm in}$ does not vanish in the nonchiral setup, in contrast to the chiral setup. We show this by investigating the mean photon number involved in the photoassisted scattering. They fulfill

$$\begin{aligned} \langle n(\mathcal{E})\rangle_{RL} + \langle n(\mathcal{E})\rangle_{LL} = &\sum_n n|a_n|^2 \, |t_{\rm st}(\mathcal{E}_n)|^2 \\ &+ \sum_{n,m,l} n\{a_m a^*_{-n+m} r_{\rm st}(\mathcal{E}_m)\}^* \, a_l a^*_{-n+l} \, r_{\rm st}(\mathcal{E}_l) \, . \end{aligned} \tag{S35}$$

$$\langle n(\mathcal{E})\rangle_{LR} + \langle n(\mathcal{E})\rangle_{RR} = 0 \, . \tag{S36}$$

The last term of Eq. (S35) is the result of interferences of all possible processes in which, e.g., an electron from the left reservoir first absorbs m photons, reflects at the resonance level, and absorbs $m-n$ photons. The appearance of such term, contrarily to the case of chiral conductor, is key factor for finite AC power injection in the nonchiral setup.

To clarify the meaning of the last term of Eq. (S35), we change the variable n to a new variable $p \equiv -n + m$ in the last term in Eq. (S35),

$$\begin{aligned} \langle n(\mathcal{E})\rangle_{RL} + \langle n(\mathcal{E})\rangle_{LL} = &\sum_n n|a_n|^2 \, |t_{\rm st}(\mathcal{E}_n)|^2 \\ &- \sum_{p,m,l} p \, a_p a^*_{p+l-m} a^*_m a_l \, r^*_{\rm st}(\mathcal{E}_m) \, r_{\rm st}(\mathcal{E}_l) \\ &+ \sum_{p,m,l} m \, a_p a^*_{p+l-m} a^*_m a_l \, r^*_{\rm st}(\mathcal{E}_m) \, r_{\rm st}(\mathcal{E}_l) \, . \end{aligned} \tag{S37}$$

For the last term, we use

$$\sum_p a_p a^*_{p+l-m} = \delta_{m,l}. \tag{S38}$$

This property follows from the fact that the left hand side is the $(m-l)$-th Fourier coefficient of $e^{i\phi_{\rm ac}(t)} e^{-i\phi_{\rm ac}(t)} = 1$. Using Eq. (S38) the last term and the first term of the

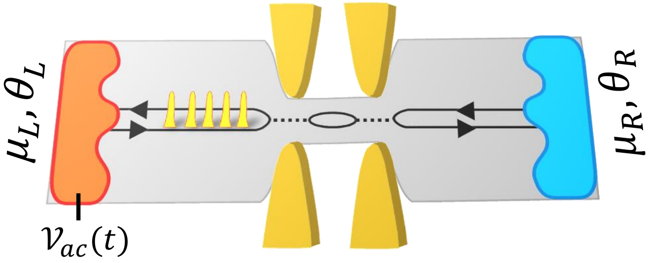

Supplementary Figure 5. Setup of nonchiral conductor. In contrast to the chiral conductor, the left-going and right-going modes are not spatially separated.

right hand side of Eq. (S37) vanish due to $\langle n \rangle = 0$. Then, we obtain

$$\langle n(\mathcal{E})\rangle_{RL} + \langle n(\mathcal{E})\rangle_{LL} = -\sum_{p,m,l} p\, a_p a^*_{p+l-m} \times a^*_m a_l\, r^*_{\rm st}(\mathcal{E}_m)\, r_{\rm st}(\mathcal{E}_l)\,. \tag{S39}$$

We utilize the fact that [$a(t)$ is the Fourier transform of the phototransition probabilities, see Eq. (S7)]

$$\sum_p p\, a_p a^*_{p+l-m} = \frac{i}{2\pi}\int_0^{2\pi/\Omega} dt\, \frac{\partial a}{\partial t} a^*(t) e^{i(m-l)\Omega t} = \frac{1}{2\pi}\int_0^{2\pi/\Omega} dt\, \frac{e\mathcal{V}_{\rm ac}(t)}{\hbar} e^{i(m-l)\Omega t}\,. \tag{S40}$$

The first equality can be verified when employing the definition of $a(t)$, Eq. (S7). In the second equality, Eq. (S10) is used. Using Eqs. (S39), (S40), we obtain the mean number

$$\langle n(\mathcal{E})\rangle_{RL} + \langle n(\mathcal{E})\rangle_{LL} = -\frac{1}{2\pi}\int_0^{2\pi/\Omega} dt\, \frac{e\mathcal{V}_{\rm ac}(t)}{\hbar} |\psi_r(t;\mathcal{E})|^2, \tag{S41}$$

where $\psi_r(t;\mathcal{E}) \equiv \sum_l a_l r_{\rm st}(\mathcal{E}_l) e^{-il\Omega t}$ is the wave function immediately after the reflection at time t for an electron of initial energy $\mathcal{E}$. The mean number of Eq. (S41) is generally nonzero and contributes to the AC power injection as explicitly demonstrated for the slow driving and zero temperature limits.

In the slow driving regime, $\hbar\Omega \ll \Gamma$, we further simplify Eq. (S41). Expanding the wave function in the driving frequency with $r_{\rm st}(\mathcal{E}_l) = r_{\rm st}(\mathcal{E}) + (\partial r_{\rm st}/\partial\mathcal{E}) l\hbar\Omega$, we find

$$\psi_r(t;\mathcal{E}) = a(t) r_{\rm st}(\mathcal{E}) + i\hbar \frac{\partial r_{\rm st}}{\partial \mathcal{E}} \frac{\partial a}{\partial t}. \tag{S42}$$

The first term of Eq. (S42) does not contribute to the mean number $\langle n(\mathcal{E})\rangle_{RL} + \langle n(\mathcal{E})\rangle_{LL}$, as the contribution is proportional to $\int_0^{2\pi/\Omega} dt\, \mathcal{V}_{\rm ac}(t)|a(t)|^2 = \int_0^{2\pi/\Omega} dt\, \mathcal{V}_{\rm ac}(t) = 0$. We now consider the second term,

$$\langle n(\mathcal{E})\rangle_{RL} + \langle n(\mathcal{E})\rangle_{LL} = -\frac{1}{2\pi}\int_0^{2\pi/\Omega} dt\, e\mathcal{V}_{\rm ac}(t) \times 2{\rm Re}\Big[i r^*_{\rm st}(\mathcal{E}) \frac{\partial r_{\rm st}}{\partial \mathcal{E}} a^*(t) \frac{\partial a}{\partial t}\Big]. \tag{S43}$$

Using Eq. (S10), we obtain

$$\langle n(\mathcal{E})\rangle_{LL} + \langle n(\mathcal{E})\rangle_{RL} = -\frac{\partial |r_{\rm st}(\mathcal{E})|^2}{\partial \mathcal{E}} \frac{e^2\overline{\mathcal{V}^2_{\rm ac}(t)}}{\hbar\Omega}. \tag{S44}$$

In the zero-temperature limit, $\theta_L \ll \Gamma$, we obtain a simple expression of the AC power injection, using Eq. (10) and (S44),

$$\overline{P_{\rm in}} = |t_{\rm st}(\mu_L)|^2 \frac{e^2}{h} \overline{\mathcal{V}^2_{\rm ac}(t)} \tag{S45}$$

This is the AC power determined by Joule's law with conductance $|t_{\rm st}(\mu)|^2 e^2/h$. It shows that the AC power injection is generally nonzero for nonchiral setups, in contrast to the chiral setup. One should not apply Eq. (S45) with $|t_{\rm st}(\mu)|^2 = 1$ to the situation when there is no resonance level, because the Floquet scattering matrix becomes different, as $\mathcal{S}_{RL}(\mathcal{E}_n,\mathcal{E}) = a_n$, $\mathcal{S}_{LR}(\mathcal{E}_n,\mathcal{E}) = a^*_{-n}$, $\mathcal{S}_{LL}(\mathcal{E}_n,\mathcal{E}) = \mathcal{S}_{RR}(\mathcal{E}_n,\mathcal{E}) = 0$, which leads to $\overline{P_{\rm in}} = 0$.

Supplementary Figure 6 shows the efficiency decrease by the AC driving in the case of nonchiral conductors. The parameters such as AC driving protocol, temperatures θ_L and θ_R are the same as in Fig. 2.

DISCUSSION ON RESERVOIRS WITH DIFFERENT CHEMICAL POTENTIALS

Here we discuss another reservoir configuration, different from the one considered in the main text, but possible in experimental situations, where each reservoir can have arbitrary chemical potentials, see Supplementary Figure 7. Below we show that any difference in the chemical potentials between the left two reservoirs diminishes the power generation as the potential bias between the two induces a power dissipation. This justifies our choice for the reservoir configuration in Fig. 1(b).

As in the main text, we employ the Floquet scattering formalism to calculate the currents into all the terminals. The Floquet scattering matrix represented in the reservoir indexes ordered as L_1, L_2, and R is

$$\mathcal{S}_{\alpha\beta}(\mathcal{E}_n,\mathcal{E}) = \begin{bmatrix} 0 & a^*_{-n} & 0 \\ a_n r_{\rm st}(\mathcal{E}_n) & 0 & \delta_{n0} t'_{\rm st}(\mathcal{E}) \\ a_n t_{\rm st}(\mathcal{E}_n) & 0 & \delta_{n0} r'_{\rm st}(\mathcal{E}) \end{bmatrix}. \tag{S46}$$

The element $S_{L_1L_2}(\mathcal{E}_n,\mathcal{E})$ is a^*_{-n} because the electron enters into the region under the AC voltage (see Section PHOTOTRANSITION AMPLITUDE a_n in SI). This

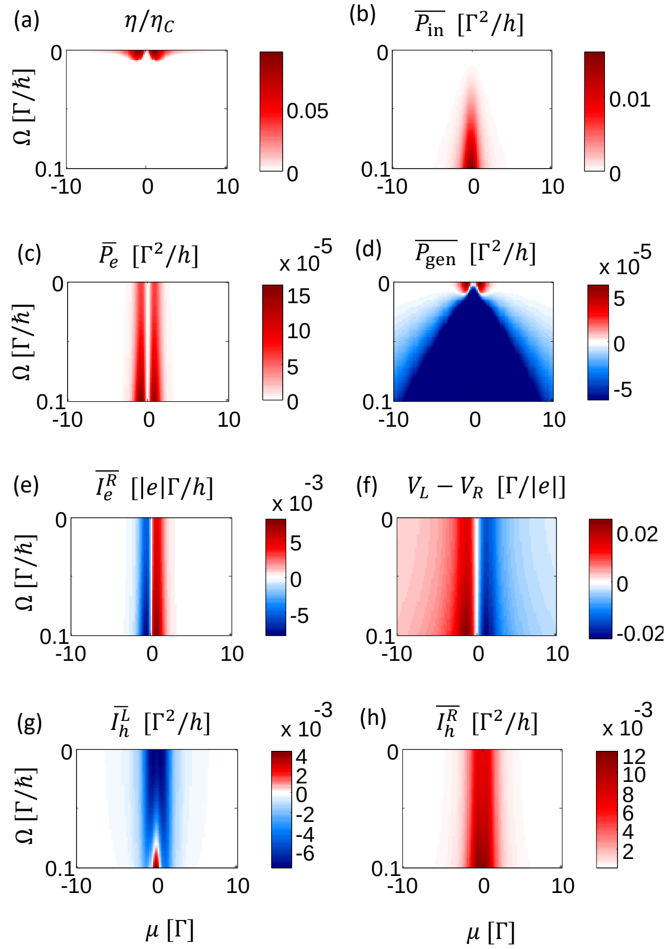

Supplementary Figure 6. Heat engine efficiency in the AC driven nonchiral conductor of Supplementary Figure 5. (a) The efficiency decreases when the AC frequency increases, satisfying the Carnot limit, in contrast to the chiral-conductor. This is due to large positive AC power injection (b) which dominates the electric power (c) and makes the generated power (d) negative. Here, the generated power lower than $5 \times 10^{-5}\Gamma^2/h$ is plotted with blue color. Additional details are also shown, such as charge current (e), DC voltage maximizing electric power chosen as in Eq. (S29) (f), and heat current into the left (g), and right (h) reservoir.

term does not contribute to the net heat and charge current, $\overline{I_h^L} = \overline{I_h^{L_1}} + \overline{I_h^{L_2}}$, $\overline{I_h^R}$, and $\overline{I_e^R}$. We note that Eq. (S46) satisfies the unitarity conditions, $\sum_{n\alpha} |S_{\alpha\beta}(\mathcal{E}_n, \mathcal{E})|^2 = 1$ and $\sum_{n\beta} |S_{\alpha\beta}(\mathcal{E}, \mathcal{E}_{-n})|^2 = 1$.

The charge, heat, and energy currents into reservoir α are determined by Eq. (S46) and Eqs. (4)– (6). The power injection by AC voltage is obtained using the energy conservation relation, $\overline{P_{\rm in}} = \sum_\alpha \overline{I_u^\alpha}$,

$$\overline{P_{\rm in}} = \int \frac{d\mathcal{E}}{h} \sum_{\alpha,\beta,n} n\hbar\Omega |S_{\alpha\beta}(\mathcal{E}_n, \mathcal{E})|^2 f_\beta(\mathcal{E}). \tag{S47}$$

This vanishes due to the chirality manifested in Eq. (S46),

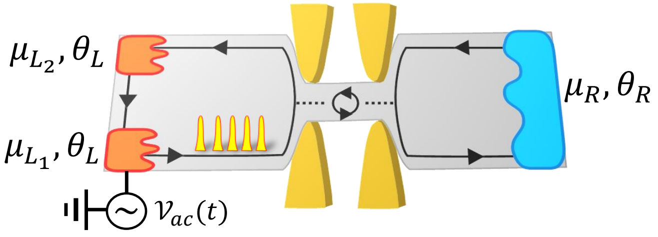

Supplementary Figure 7. Three terminal setup with arbitrary chemical potentials for each reservoir.

$\sum_n |a_n|^2 = 1$, and $\sum_n n|a_n|^2 = 0$,

$$\overline{P_{\rm in}} = 0. \tag{S48}$$

The electric power generated by the charge flow is

$$\overline{P_e} = \sum_\alpha \mu_\alpha \overline{I_e^\alpha}/e. \tag{S49}$$

Using the form of the Floquet scattering matrix, Eq. (S46), we obtain the electric power,

$$\begin{aligned}\overline{P_e} &= (\mu_R - \mu_{L_2})\frac{\overline{I_e^R}}{e} \\ &+ (\mu_{L_1} - \mu_{L_2}) \int \frac{d\mathcal{E}}{h}\Big(- f_{L_1}(\mathcal{E}) + f_{L_2}(\mathcal{E})\Big),\end{aligned} \tag{S50}$$

The second term is a nonpositive quantity which describes the power dissipation between the reservoirs L_1 and L_2. It vanishes when the two reservoirs have the same chemical potential.

Therefore, the condition used in the main text, $\mu_{L_1} = \mu_{L_2}$, is the best choice for obtaining large electric power. Defining $\mu_L \equiv \mu_{L_1} = \mu_{L_2} \equiv$ and $f_L(\mathcal{E}) \equiv f_{L_1}(\mathcal{E}) = f_{L_1}(\mathcal{E})$, the electric power $\overline{P_e}$, the total heat current into the hot reservoir $\overline{I_h^L} \equiv \overline{I_h^{L_1}} + \overline{I_h^{L_2}}$, and the efficiency become equal to the quantities described in the main text.
